# Fangchinoline (2021–2026): A Multitarget Bisbenzylisoquinoline Alkaloid With Emerging Anti‐Infective Potential

**DOI:** 10.1155/jotm/5617306

**Published:** 2026-09-15

**Authors:** Alaa Sirwi, Hagar M. Mohamed, Gamal A. Mohamed, Basma G. Eid, Hossam M. Abdallah, Sabrin R. M. Ibrahim

**Affiliations:** ^1^ Department of Natural Products and Alternative Medicine, Faculty of Pharmacy, King Abdulaziz University, Jeddah 21589, Saudi Arabia, kau.edu.sa; ^2^ Animal House Unit, King Fahd Medical Research Center, King Abdulaziz University, Jeddah 22254, Saudi Arabia, kau.edu.sa; ^3^ Department of Medical Laboratory Analysis, College of Medical & Health Sciences, Liwa University, Abu Dhabi 41009, UAE; ^4^ Department of Applied Medical Chemistry, Medical Research Institute, Alexandria University, Alexandria, Egypt, alexu.edu.eg; ^5^ Department of Pharmacology and Toxicology, Faculty of Pharmacy, King Abdulaziz University, Jeddah 21589, Saudi Arabia, kau.edu.sa; ^6^ Preparatory Year Program, Department of Chemistry, Batterjee Medical College, Jeddah 21442, Saudi Arabia, bmc.edu.sa; ^7^ Department of Pharmacognosy, Faculty of Pharmacy, Assiut University, Assiut 71526, Egypt, aun.edu.eg

**Keywords:** antiviral activity, autophagy modulation, bisbenzylisoquinoline alkaloid, drug repurposing, fangchinoline, hepatoprotection, infectious diseases, PI3K/AKT/mTOR signaling

## Abstract

**Background:**

Fangchinoline (FC) is a bisbenzylisoquinoline alkaloid reported mainly from the traditional Chinese medicinal plant, *Stephania tetrandra*. FC has garnered scientific attention because of its wide‐ranging biological properties and multitargeted mechanisms of action. This review summarizes the reported studies on the pharmacological activities of FC from 2021 to August 2026, with particular attention to its anti‐infective activities and associated mechanisms.

**Methods:**

A thorough literature search was conducted through various databases, including ScienceDirect, Web of Science, Scopus, and Google Scholar, together with publishers’ websites (e.g., Elsevier, Wiley, Springer, Taylor & Francis, Sage, Thieme, and Bentham) from 2021 to August 2026.

**Results:**

Recent studies have investigated the activity of FC against several viral and microbial pathogens using different experimental models. Across these studies, the reported antiviral mechanisms included interference with viral entry and internalization, alteration of endolysosomal trafficking and autophagy, and modulation of innate immune signaling. Antimicrobial studies reported activity against *Candida albicans* and intracellular *Salmonella*, with enhancement of lysosomal function reported in the latter model. FC has also demonstrated hepatoprotective, antifibrotic, antidiabetic, anti‐inflammatory, and other biological activities in preclinical models.

**Conclusion:**

Collectively, these findings demonstrate the potential of FC as a multitarget natural scaffold with reported antiviral, antimicrobial, and other pharmacological activities; however, the evidence remains predominantly preclinical. Further studies are required to clarify its mechanisms of action and establish its pharmacokinetic, bioavailability, and safety profiles and to determine whether the reported anti‐infective activities can be translated into clinically relevant applications.

## 1. Introduction

Medicinal plants have been studied since ancient times because of their effectiveness in preventing and treating various illnesses, with favorable patient acceptance and tolerance [1]. Effective plants have been chosen through simple trial and error, and this knowledge has been transmitted verbally or mystically from one generation to the next [2]. It is estimated that plants are the source of about 25% of the available medications [3, 4].

**FIGURE 1:**
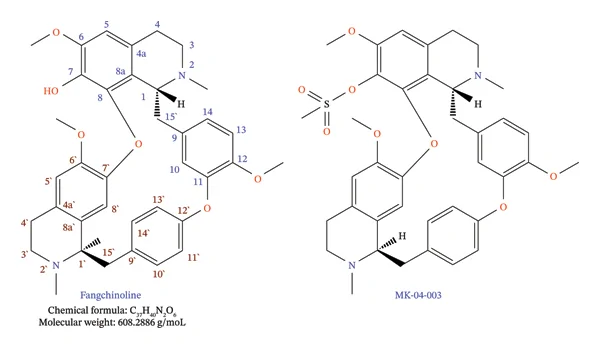
Chemical structure of fangchinoline (FC) and its derivative MK‐04‐003.

*Stephania tetrandra* is an alkaloid‐rich medicinal plant prevalent in the subtropical and tropical regions of Africa and Asia. Its roots, known as “Fen Fang Ji,” have been used in traditional Chinese medicine for treating rheumatism‐related arthralgia, dysuria, wet beriberi, inflamed sores, eczema, dysentery, asthma, malaria, fever, hypertension, cancer, hyperglycemia, and tuberculosis [5, 6]. Phytochemical investigations showed that bisbenzylisoquinoline alkaloids (BBAs) are prominent constituents of *S. tetrandra* roots [7]. Fangchinoline (FC; molecular formula C_37_H_40_N_2_O_6_, and molecular weight 608.29) is one of the main BBAs isolated from *S*. *tetrandra* roots [8]. FC consists of two benzylisoquinoline units connected by oxygen bridges at C8–C7′ and C11–C12′. It has nitrogen atoms at C2 and C2′, OCH_3_ groups at C6, C12, and C6′, and a hydroxyl function at C7 [6, 9] (Figure 1). Physicochemically, FC is described as a white to light‐yellow powder with a melting point of 240°C–242°C and pKa value of 9.38 ± 0.20. It is slightly soluble in water and soluble in chloroform, methanol, and DMSO [10, 11].

Quantitative studies on *S. tetrandra* roots reported average FC contents (1.87 mg/g and 0.40%, respectively) using nonaqueous capillary electrophoresis [12] and liquid chromatography with electrochemical detection, respectively [13]. FC (concentration from 2.9 to 14.3 g/kg) was found to be a key component of Qifang Xibi Granule, a Chinese remedy for osteoarthritis (OA) [14]. Additionally, FC was separated from other *Stephania* species, including *S. hernandifolia*, *S. cepharantha*, *S. rotunda*, and *S. japonica* [15]. FC has attracted considerable interest because of preclinical research, revealing diverse bioactivities, including anti‐infective and anticancer activities mediated through multiple cellular and molecular mechanisms [8]. Previously published reviews by Mérarchi et al. and Chan et al. focused largely on the anticancer properties of FC, including cancer‐related molecular targets, signaling pathways, and multidrug resistance [7, 8]. Since these reviews, increasing evidence has also examined the activity of FC against viral and microbial pathogens, broadening interest in its pharmacological profile beyond oncology. Considering the rapid increase in published studies on FC since Chan et al., the current review aims to summarize the published research from 2021 to August 2026, with a particular emphasis on its anti‐infective activities and associated mechanisms, while also discussing other reported pharmacological effects, pharmacokinetic (PK) characteristics, and translational limitations.

## 2. Search Methodology

**FIGURE 2:**
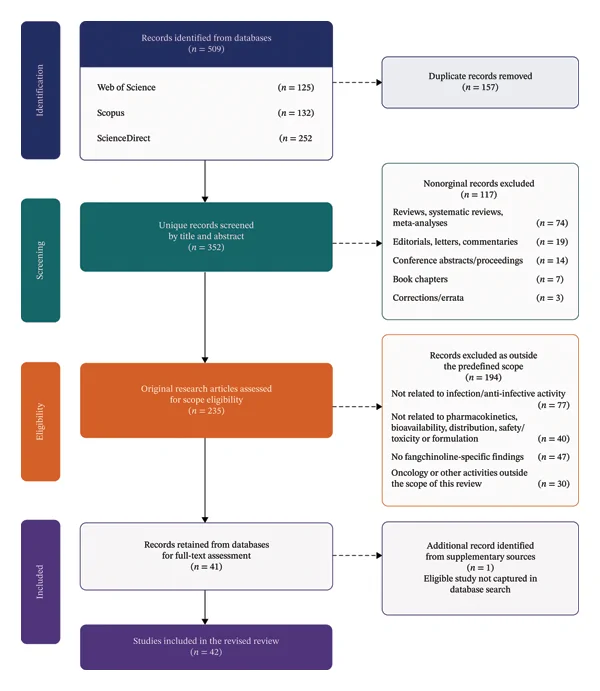
Flow diagram of the literature search and study selection process.

A structured literature search was conducted using Scopus, Web of Science, and ScienceDirect to identify relevant publications from January 2021 to August 2026, using “fangchinoline” alone and in combination with terms related to the scope of the review, including “infection,” “infectious disease,” “antiviral,” “antibacterial,” “antimicrobial,” “antifungal,” “anti‐inflammatory,” “antioxidant,” “pharmacokinetics,” “bioavailability,” “toxicity,” and “safety.” Records retrieved from the principal databases were compiled, and duplicates were removed. Google Scholar and reference lists of relevant articles were additionally searched to identify potentially relevant literature. Titles and abstracts were screened for relevance to the review scope, followed by the full‐text assessment of potentially eligible publications. Original research articles reporting FC‐specific findings related to infectious diseases or anti‐infective activity, including antiviral, antibacterial/antimicrobial, and antifungal effects, as well as anti‐inflammatory or antioxidant effects, were considered for inclusion. Original studies providing FC‐specific data on PKs, bioavailability, distribution, safety/toxicity, or herbal formulations were also considered because of their relevance to translational assessment. In addition, original studies reporting other pharmacological activities of FC were included in the review and were considered eligible when they provided relevant mechanistic or preclinical evidence. Records outside the defined publication period, duplicate publications, conference abstracts, editorials, reviews, book chapters, corrections/errata, fully written in a non‐English language, studies lacking FC‐specific findings, and original studies addressing oncology were excluded. Studies published before January 2021 were not included in the defined search period but were cited where necessary to provide relevant background. The literature identification, screening, and selection process is summarized in the flow diagram in Figure 2.

## 3. Biological Activities of FC

Recent studies have investigated the activity of FC against several viral and microbial pathogens using different experimental models. Across these studies, the reported mechanisms included interference with viral entry and internalization, alteration of endolysosomal trafficking and autophagy, modulation of innate immune signaling, and enhancement of lysosomal function. The available evidence is discussed below according to the infectious agent, experimental model, and reported mechanism.

### 3.1. Antiviral Activity

**FIGURE 3:**
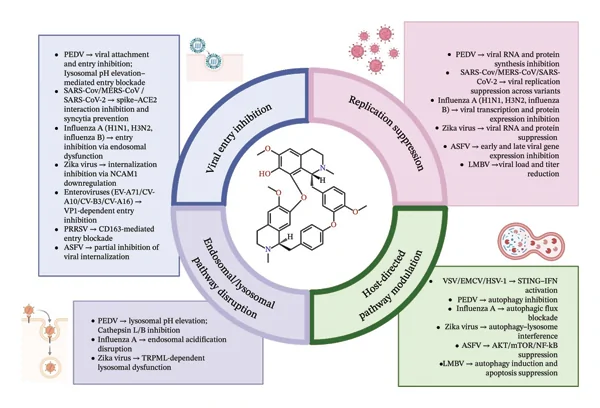
Summary of the reported mechanisms of FC antiviral activity.

Additionally, several studies reported the broad in vitro and in vivo antiviral capacity of FC against various viruses through diverse mechanisms, which are discussed in Table 1 (Figure 3).

**TABLE 1:** Antiviral activity of fangchinoline across reported studies.

Activity	Study type	Experimental model	Main findings	Ref.
Antiflavivirus	In vitro	A549, Vero, BHK‐21, RD cells Viruses: ZIKV, DENV‐2, JEV	Bis‐benzylisoquinoline alkaloids (BBAs) significantly reduced flavivirus infection in various cell lines by blocking viral entry and genome replication BBAs caused lysosomal alkalization, TRPML channel blockade, and ¯ NCAM1 expression, making cells less susceptible. BBAs inhibited autophagy fusion, leading to the accumulation of autophagosomes and suppression of viral RNA replication	[16]

Anti‐Zika virus	In vitro and in vivo	BHK‐21 cells; ZIKV‐infected suckling mice	In BHK cells, FC showed potent inhibition of ZIKV (EC_50_ 0.86 μM, CC_50_ 8.4 μM, SI 9.83) FC prevented ZIKV cytopathic effects and reduced viral RNA FC (4 mg/kg IP) improved survival to 60% (vs 0% in controls), maintained body weight, and lowered viral loads in blood and tissues in neonatal mice	[17]

Anti‐influenza A virus	In vitro	MDCK and A549 cells Influenza A H1N1 and H3N2 (including resistant clinical isolates)	FC inhibited H1N1 and H3N2 replication in a dose‐dependent manner FC suppressed viral PB2 mRNA/protein levels (> 80% at the maximum concentration) and ¯M2 protein signal, as determined by immunofluorescence FC inhibited oseltamivir‐resistant (H1N1) and amantadine‐resistant (H3N2) strain replication FC activity in the early stages by preventing the endosomal pH drop required for viral uncoating	[18]

Anti‐H1N1 influenza	In vitro	MDCK cells, A549 cells H1N1 influenza	FC ¯ H1N1 viral RNA and NP protein levels in MDCK cells and protected cells from virus‐induced death FC induced LC3 accumulation and LC3‐II increase, indicating autophagosome buildup FC prevented autophagosome/lysosome fusion, causing virions to be sequestered in autophagosomes and blocking infection	[19]

Anti‐pan‐β‐coronavirus	In vitro and in vivo	Vero‐E6 and other cell lines (SARS‐CoV‐2, SARS‐CoV, MERS‐CoV); hACE2‐transgenic mice; Syrian hamsters (SARS‐CoV‐2 infection)	FC inhibited replication of SARS‐CoV, MERS‐CoV, SARS‐CoV‐2 and its variants (B.1.1.529, B.1.617.2, B.1.1.7, XBB.1, and BA.5) FC synergistically inhibited SARS‐CoV‐2 replication in combination with clofazimine and remdesivir	[20]

Antiviral via immunomodulation	In vitro and in vivo	Multiple viruses (VSV, EMCV, H1N1, HSV‐1); viral sepsis (mice)	FC suppressed HSV‐1, EMCV, and H1N1 replication in both mouse and human cells FC improved survival in a mouse viral sepsis model by prolonging Type I interferon signaling FC elicited antiviral immunity via STING activation	[21]

Anti‐influenza viruses	In vitro	MDCK and A549 cells; influenza A H1N1 and H3N2 (including resistant clinical isolates)	FC inhibited viral replication in MDCK cells (CPE inhibition: IC_50_ 4.96 μM for H1N1; 4.45 μM for H3N2) FC suppressed viral PB2 mRNA and protein expression and reduced M2 protein expression in MDCK and A549 cells FC (10 μM; > 70% suppression) suppressed oseltamivir‐resistant (H1N1) and amantadine‐resistant (H3N2) strains	[18]

Anti‐PEDV	In vitro	Vero cells infected with porcine epidemic diarrhea virus (PEDV)	FC dose‐dependently inhibited PEDV virus (CC_50_ 30.19 μM, IC_50_ 6.69 μM, SI 4.51) FC interfered with virus entry/attachment or directly attenuated the virus	[22]

Anti‐PEDV	In vitro	Cell‐based PEDV infection assays (Vero‐E6 cells)	FC suppressed PEDV (IC_50_ 3.54 μM; CC_50_ 17 μM; SI 4.8) FC ¯ cathepsin L and B activity by suppressing lysosome acidification, resulting in the inhibition of PEDV entry	[23]

Anti‐PEDV	In vitro	IPEC‐J2 intestinal epithelial cells infected with PEDV	FC dose‐dependently inhibited PEDV infection (EC_50_ 0.67 μM) FC blocked autophagic flux in PEDV‐infected cells	[24]

Anti‐TGEV	In vitro	TGEV‐infected PK‐15 and IPEC‐J2 cells	FC inhibited TGEV replication (EC_50_ = 2.862 and 3.670 μM, respectively) FC interfered with viral attachment, internalization, and later replication stages	[25]

Anti‐African swine fever virus (ASFV)	In vitro	Porcine alveolar macrophages (PAMs) infected with ASFV	FC prohibited ASFV replication in PAMs (IC_50_ 1.66 μM; CC_50_ 34.6 μM; SI 20.8) FC ¯ viral protein expression and virus titer FC suppressed the ASFV‐induced activation of the AKT/mTOR/NF‐κB pathway FC inhibited IL‐1β and TNF‐α upregulation	[10]

Anti‐African swine fever virus (ASFV)	In vitro study	Porcine alveolar macrophages (PAMs) infected with ASFV	FC inhibited ASFV in PAMs (IC_50_ 0.62 μM; SI 73.61) FC suppressed the ASFV B646L gene transcription	[26]

Anti‐enteroviruses (EV‐A71; HFMD‐associated CV‐A10, CV‐B3, CV‐A16)	In vitro screening	Vero cells infected with EV‐A71; enterovirus strains (CV‐A10, CV‐B3, CV‐A16)	FC inhibited EV‐A71 (EC_50_ 0.9 μM; CC_50_ 21.14 μM; SI 23.6) FC ¯ viral titers by 600‐fold at 10 μM FC inhibited CV‐A10, CV‐B3, and CV‐A16	[27]

#### 3.1.1. Flavivirus Inhibition

Flaviviruses such as Japanese encephalitis (JEV), dengue (DENV), and Zika (ZIKV) pose a major public health challenge, with no approved specific treatments currently available [16]. In the search for antiviral agents, Huang et al. examined the activity of BBAs, including cepharanthine, berbamine, iso‐tetrandrine, tetrandrine, and FC against flaviviruses. These compounds effectively inhibited JEV, ZIKV, and DENV infections by blocking the genome replication and virus entry stages [16]. They were shown to target endolysosomes, elevating lysosomal pH (alkalization) and disrupting normal endosomal trafficking [16]. In ZIKV‐infected cells, these alkaloids blocked the endolysosomal Ca^2+^ channel TRPML, resulting in lysosomal dysfunction and reduced expression of the ZIKV entry receptor NCAM1, reducing the susceptibility of the cells to ZIKV infection [16]. Additionally, they suppressed autophagic flux and prevented autophagosome–lysosome fusion, leading to the accumulation of autophagosomes and a significant reduction in viral RNA replication [16]. Collectively, these compounds possessed antiviral capacities against flaviviruses mediated by interference with endolysosomal entry pathways [16].

An additional study by Yang et al. focused on FC that demonstrated potent in vitro and in vivo anti‐ZIKV potential. In BHK cells, FC inhibited ZIKV (EC_50_ 0.86 μM with minimal cytotoxicity (CC_50_ 8.4 μM; SI [selectivity index] 9.83) [17]. FC dose‐dependently protected cells from ZIKV‐induced cytopathic effects, inhibited ZIKV RNA replication, and reduced the production of infectious virus particles without notable toxicity [17]. In vivo, FC (4 mg/kg/i.p.) in ZIKV‐infected neonatal mice remarkably improved survival and mitigated the signs of disease. FC‐treated mice exhibited lower blood and tissue viral loads and preserved tissue integrity in the brain and kidney compared to the controls [17]. Dosing‐time assays revealed that FC exerted its action at an early stage of infection, mainly by impeding ZIKV internalization into host cells [17]. These studies established BBAs, especially FC, as potential inhibitors of flavivirus entry and replication via interference with host endolysosomal pathways and autophagy [16, 17].

#### 3.1.2. Beta‐Coronaviruses

Sadhu et al. reported that FC has anticoronavirus activity against multiple coronaviruses, including severe acute respiratory syndrome coronavirus (SARS‐CoV), Middle East respiratory syndrome coronavirus (MERS‐CoV), and severe acute respiratory SARS‐CoV‐2, by blocking viral entry into host cells [20]. FC dramatically reduced infection by these coronaviruses at sub‐micromolar concentrations in vitro [20]. In vivo, FC protects against SARS‐CoV‐2 in human ACE2‐transgenic mice and Syrian hamster infection models, where it lowered lung viral titers and diminished lung inflammation. Moreover, combination therapy of FC with remdesivir or clofazimine revealed synergistic effects, leading to stronger viral suppression than the drug alone. The synthetic FC analog (MK‐04‐003) possessed more effective inhibition of SARS‐CoV‐2 and its variants (BA.5 and B.1.617.2) than FC. These findings suggested FC as a pan‐coronavirus entry inhibitor, and further studies on FC and its analogs are required for preclinical development [20].

Khadilkar et al. evaluated FC, tetrandrine, and cepharanthine against SARS‐CoV‐2 using cell‐based infection assays [28]. FC inhibited wild‐type SARS‐CoV‐2 infection in 293T cells expressing ACE2 and TMPRSS2, and it also showed activity against the Delta variant. However, the antiviral effect differs according to the cell model, as FC did not inhibit wild‐type SARS‐CoV‐2 infection in Vero cells expressing ACE2 and TMPRSS2. The artificial membrane permeability assay revealed that these alkaloids are biologically available, whereas FC showed lower permeability than tetrandrine [28]. Phospholipidosis was also observed particularly in 293T cells at higher concentrations of these alkaloids. These findings demonstrated the effect of the experimental model and concentration on the observed antiviral activity that should be taken in consideration when interpreting these findings′ translational relevance [28]. Computational studies have also explored the potential interactions of FC with SARS‐CoV‐2 proteins. Rani et al. identified FC among seven compounds having predicted binding to all four major viral structural proteins‐spike (S), nucleocapsid (N), membrane (M), and envelope (E). Among the related BBAs, FC, tetrandrine, and cepharanthine all showed multitarget binding in the initial docking analysis, while subsequent molecular dynamics analysis identified FC as a potential inhibitor of the N‐protein C‐terminal domain [29]. In another in silico study, FC showed stronger predicted binding affinity toward SARS‐CoV‐2 3CLpro than the reference inhibitor ML188 [30]. Boozari et al. similarly reported predicted binding of FC to the SARS‐CoV‐2 main protease, with a docking energy of −10.1 kcal/mol and a calculated Gibbs free binding energy of −42.26 kJ/mol compared with −30.9 kJ/mol for lopinavir [31]. However, these findings are supportive predictions based on computational approaches rather than direct evidence of SARS‐CoV‐2 inhibition.

#### 3.1.3. Porcine Reproductive and Respiratory Syndrome Virus (PRRSV)

Porcine reproductive and respiratory syndrome virus is a highly pathogenic virus that causes severe disease in swine. Unfortunately, the current vaccines are inadequately protective [32]. Zheng et al. evaluated natural compounds using structure‐based virtual screening to identify those that target the host receptor CD163. CD163 is a key entry factor that mediates PRRSV virion uncoating in pig macrophages. Among them, FC was found to effectively inhibit PRRSV by targeting the host receptor CD163. It was strongly bound to the CD163‐SRCR5 domain (binding energy −16.9 kJ/mol) and interacted with the GLY‐495 residue during molecular docking. FC suppressed viral replication in Marc‐145 cells (EC_50_ 2.03 μM; CC_50_ 14–16 μM; SI 6.90–7.88). FC (conc. 10 μM) dose‐dependently reduced PRRSV‐N protein and viral loads. FC blocked viral entry by interfering with CD163‐mediated virus–receptor interactions [32].

#### 3.1.4. Influenza, Vesicular Stomatitis (VSV), Encephalomyocarditis (EMCV), and Herpes Simplex‐1 (HSV‐1) Viruses

Influenza viruses are a significant public health concern because of their high mutation rates and development of drug resistance, and they are known to cause frequent epidemics and pandemics [33].

Li et al. identified the BBAs (cepharanthine/tetrandrine/FC/berbamine/iso‐tetrandrine) as potent inhibitors of influenza A (H1N1) replication in human lung (A549) and Madin–Darby canine kidney (MDCK) cells [18]. These compounds suppressed influenza‐induced cytopathic effects, with actions concentrated in the early stages of the viral life cycle [18]. The prevention of endosomal pH drop is needed for viral envelope fusion, thereby blocking the release of the viral ribonucleoprotein (vRNP) into the cytoplasm [18]. Xie et al. studied FC against H1N1 influenza in MDCK cells and revealed a similar mechanism involving autophagy [19]. FC (2.5–10 μM) significantly inhibited H1N1 replication in MDCK cells. FC blocked autophagic flux in infected cells. FC prevented autophagosome/lysosome fusion, trapping incoming virions in autophagosomes and preventing their replication [19]. Thus, FC interfered with the autophagy pathway by increasing endosomal/lysosomal pH and blocking autophagic maturation [18, 19]. Li et al. reported that FC demonstrated notable antiviral activity against influenza viruses through early stages of infection [18]. FC suppressed influenza A virus replication H1N1 and H3N2 (IC_50_s 4.96 and 4.45 μM; SI: 6.21 and 6.93, respectively), while its cytotoxicity values (CC_50_) were 47.71 μM against MDCK cells and 46.77 μM in A549 cells. FC (Conc. 10 μM; 70% suppression) also suppressed the replication of oseltamivir‐resistant H1N1 (A/TianjinJinnan/15/2009) and amantadine‐resistant H3N2 (A/HunanZhuhui/1222/2010). Furthermore, FC demonstrated anti‐influenza B virus activity by inhibiting HA protein expression in the B/Victoria and B/Yamagata lineages. FC further disrupted endosomal acidification by elevating late endosomes′ pH, resulting in the prevention of vRNP release into the cytoplasm [18].

Wang et al. revealed a distinct antiviral mechanism of FC via immunomodulation. FC significantly inhibited diverse viruses in vitro, including vesicular stomatitis virus, EMCV, H1N1 influenza, and HSV‐1 [21]. In a lethal viral sepsis mouse model, FC treatment reduced systemic viral loads, attenuated tissue inflammation, and improved survival [21]. FC activated innate antiviral immunity in a STING (stimulator of interferon genes)‐dependent manner, leading to elevated production of Type I interferons and interferon‐stimulated genes [21]. FC was found to bind STING and prevent its proteolytic degradation, thereby prolonging STING activation and sustaining interferon signaling [21].

Recently, Cheng et al. have examined the FC action in H1N1 infection and identified FC as an enhancer of lysosomal gene expression using connectivity map screening and transcriptomic analysis [34]. FC accumulated within the lysosomes, increased lysosomal luminal pH, and induced TFEB nuclear translocation, leading to restoring lysosomal biogenesis. It also impaired autophagic flux and disrupted autophagosome–lysosome fusion. It was shown that FC inhibited H1N1 infection at the entry stage by obstructing endo‐lysosomal trafficking. Furthermore, the regulation of the autophagy–lysosome system and its antiviral effect were found to depend on TFEB [34].

#### 3.1.5. Porcine Epidemic Diarrhea Virus (PEDV)

Dong et al. showed that FC in vitro inhibited PEDV in Vero cells (IC_50_ 6.69 μM; CC_50_ 30.19 μM; SI 4.51). FC markedly reduced viral titers, eliminated PEDV‐N protein, and suppressed M protein, suggesting the inhibition of viral replication. FC mediated its effect by interfering with the attachment processes and virus entry, or through the direct attenuation of the virus [22]. FC also suppressed PEDV activity (IC_50_ 3.54 μM) and decreased PEDV‐N protein levels and virus titers in vitro. FC suppressed lysosome acidification, resulting in reduction in cathepsins L and B activity, thereby inhibiting PEDV entry [23]. Furthermore, Zhang et al. reported that FC (EC_50_ 0.67 μM; CC_50_ 37.49 μM) dose‐dependently prohibited a PEDV infection at 24 h postinfection in IPEC‐J2 cells. It also influenced PEDV replication and inhibited PEDV at the viral life cycle internalization and attachment stages. Thus, it prevented autophagic flux in PEDV‐infected cells [24].

#### 3.1.6. Transmissible Gastroenteritis Virus (TGEV)

Hu et al. investigated the antiviral effect of FC against TGEV and reported porcine aminopeptidase N (pAPN) as a potential target [25]. FC inhibited TGEV in PK‐15 and IPEC‐J2 cells (EC_50_s 2.862 and 3.670 μM, respectively) and lowered TGEV‐N protein expression and viral RNA copies. It also suppressed the TGEV‐mediated expression of IL‐6 and TNF‐α. Furthermore, cellular thermal shift assay (CETSA) and drug affinity responsive target stability (DARTS) assays supported the interaction between FC and pAPN, and FC treatment reduced pAPN protein expression. It was found that FC affected both the early stages of TGEV infection, including attachment and internalization, and later stages of viral replication. In vitro, FC also inhibited PEDV (EC_50_ 2.243 μM) [25].

#### 3.1.7. African Swine Fever Virus (ASFV)

Su et al. reported that FC (IC_50_ 1.66 μM) significantly inhibited ASFV replication in porcine alveolar macrophages (PAMs). FC suppressed viral replication by inhibiting the ASFV‐induced activation of the AKT/mTOR/NF‐κB signaling pathway [10]. A complementary study by Zhu et al. confirmed FC activity against ASFV in PAMs (IC_50_ 0.62; SI 73.61 μM). FC significantly inhibited ASFV B646L gene transcription. It also inhibited ASFV at the entry and postentry replication stages [26].

#### 3.1.8. Enteroviruses

Zhang et al. evaluated 1042 natural compounds using an EV‐A71‐eGFP high‐content screening approach. FC was the most potent compound inhibiting EV‐A71 infection in Vero cells (conc. 10 μM; EC_50_ 0.9 μM; CC_50_ 21.14 μM; SI 23.6) with a 600‐fold decrease in viral titer. FC (EC_50_s 0.86–1.68 μM) also exhibited antienterovirus activity against HFMD‐linked viruses (CV‐A10, CV‐B3, and CV‐A16 strains). FC acted mainly at an early stage of infection without notable virucidal effects and inhibition of viral RNA translation/genome replication [27]. Interestingly, VP1 was identified as a potential target of FC, revealing FC as a promising entry‐stage antiviral agent against multiple enteroviruses [27].

The reported antiviral effects of FC involve multiple stages of viral infection, including viral entry and internalization, replication, endosomal/lysosomal pathways, and host‐directed signaling pathways, as summarized in Figure 3.

### 3.2. Antimicrobial Activity

Fungal diseases impact millions of people globally. It is estimated that more than 6.55 million people are affected yearly by life‐threatening fungal diseases, resulting in more than 3.75 million deaths, and approximately 2.55 million are directly linked to fungal diseases [35]. Their incidence has increased due to the increase in susceptible populations, late detection, drug resistance, and the lack of effective antifungal medications [36].

Yang et al. revealed that FC demonstrated antifungal capacities against many *Candida* species, including *C. krusei, C. albicans, C. tropicalis*, and *C. glabrata* (MICs 12.5–50 μg/mL) and MFCs 50–200 μg/mL, revealing its fungistatic and fungicidal properties [36]. FC also inhibited *C. albicans* virulence factors such as biofilm formation, adhesion, and hyphal growth, and it inhibited the metabolic activity of *C. albicans* biofilms. Its anti‐Candida was induced by elevating the permeability of the cell membrane and intracellular ROS overproduction. It showed notable synergistic potential with antifungal agents such as caspofungin, amphotericin B, and fluconazole, supporting further investigation of FC in combination with antifungal agents [36].

*Salmonella* has become an important organism contributing to human foodborne illnesses in the past few years [37]. Salmonella has developed complicated survival and replication mechanisms within macrophages and epithelial cells, leading to serious illnesses in both animals and humans, ranging from simple gastroenteritis to severe diseases like septicemia or typhoid fever. The development of multidrug‐resistant strains of the frequently used antibiotics such as trimethoprim–sulfamethoxazole, cephalosporins, ampicillin, and chloramphenicol represents a serious concern in terms of environmental sustainability, human well‐being, and global food security [37]. This highlights the need for new anti‐infective agents.

FC exhibited anti‐Salmonella potential by reducing the survival of *S. enterica* both in vitro and in vivo. In RAW264.7, FC decreased bacterial survival with > 90% cell viability. FC (conc. 10 mg/kg) minimized bacterial loads in the spleen, liver, cecum, colon, and ileum, reduced TNF‐α, and increased IFNg, IL‐4, and IL‐10 in S. enterica‐infected mice. FC promoted lysosomal acidification and autophagy via the AMPK–mTORC1–TFEB pathway, and it alleviated the inflammatory response in Salmonella‐infected cells [38].

Sadhu et al. identified FC as a host‐directed immunomodulator against intracellular enteric pathogens [39]. FC restricted intracellular *S. Typhi* and *S. Typhimurium* through NOS2‐dependent NO signaling, with its antibacterial activity markedly reduced following inhibition or loss of NOS2. In vivo, FC reduced bacterial burden and improved survival in *S. Typhimurium*‐infected wild‐type mice. FC also enhanced the efficacy of suboptimal dose ciprofloxacin and restricted multidrug‐resistant *Salmonella* and *Shigella flexneri* [39].

### 3.3. Hepatoprotective and Antifibrotic Activities

Hepatic injury, including both acute ischemic injury and progressive fibrotic damage, remains a significant clinical challenge.

Li et al. investigated the protective effect of FC (10 mg/kg/orally/5 days) against hepatic ischemia/reperfusion injury in rats [40]. FC was found to significantly lower serum hepatic marker enzymes (lactate dehydrogenase [LDH], ALT, and AST) and inflammatory (IL‐8, IL‐6, and TNF‐α) markers, while boosting antioxidants (CAT, GSH‐Px, and SOD) and minimizing MDA levels in serum and liver tissues. FC inhibited TNF‐α and IL‐6 with improved binding affinity in molecular docking, suggesting that FC safeguarded against ischemia/reperfusion‐linked liver damage by minimizing oxidative stress and inflammation [40].

Recently, Yin et al. have reported that FC prevented diethylnitrosamine‐induced hepatic fibrosis in mice. FC slowed histopathological modifications, suppressed collagen deposition, and blocked extracellular matrix formation and activation of hepatic stellate cells [41]. It reversed the changes induced by diethylnitrosamine in taurine levels and the enzymes that accelerate taurine formation. These findings supported the fact that FC inhibited the development of fibrosis by regulating oxidative stress and taurine‐related metabolism [41]. Recently, Du et al. further identified FC’s antifibrotic capacity against pancreatic fibrosis [42]. In a caerulein‐induced mouse model of chronic pancreatitis, FC (2.5 and 5 mg/kg, i.p.) alleviated atypical hyperplasia of metaplastic ducts and pancreatic fibrosis, with reductions of 64.3% and 70.8%, respectively. FC suppressed downstream FAK/ZEB1 signaling and targeted SRC and ERBB2, associated with reversal of EMT and reduced profibrotic responses [42].

### 3.4. Neuroprotective Activity

Alzheimer’s disease (AD) is the most predominant neurodegenerative illness in elderly people, affecting more than 50 million individuals globally [43]. FC possessed neuroprotective potential, particularly in AD. Yi et al. reported that FC improved AD‐linked disorders through the enhancement of antioxidant defenses and autophagy [43]. FC lowered amyloidogenic sequences of APP by enhancing the autophagic removal of BACE1 (β‐site APP‐cleaving enzyme‐1) (an enzyme that initiates Aβ formation) and autophagic flux in transfected N2a neuroblastoma cells. It remarkably improved memory and learning performance in a mouse model, which demonstrated increased endogenous antioxidants and inhibited oxidative stress [43]. Thus, FC’s neuroprotective potential is through the induction of autophagy and mitigation of oxidative stress, suggesting its potential for AD [43].

### 3.5. Antiglaucoma Activity

Glaucoma is a slowly progressing eye problem that may ultimately lead to blindness. It has been linked to increased intraocular pressure and progressive loss of retinal ganglion cells. Li et al. reported that FC (10 and 30 mg/kg/i.p/1 week) notably lowered the intraocular pressure in a unilateral laser‐induced ocular hypertension mouse model [44]. FC enhanced vitreous humor glutamate levels, alleviated oxidative stress in the aqueous humor, decreased retinal inflammatory cytokine levels and microglia counts, and increased retinal ganglion cell numbers. This suggested that FC exhibited neuroprotective effects in experimental glaucoma by aligning the reduction in intraocular pressure with concurrent oxidative stress and neuroinflammation mitigation [44].

### 3.6. Antidiabetic Activity

FC showed protective capacity against metabolic disorders, mainly through antioxidative mechanisms (Table 2) [45]. FC (100 or 200 mg/kg/oral/daily/45 days) remarkably attenuated diabetic oxidative stress and complications in streptozotocin‐induced diabetes in rats. Additionally, it markedly reduced excessive lipid peroxidation in the heart, serum, kidney, and liver tissues, restoring MD levels, and ROS levels [45]. It improved antioxidant defense by increasing the activities of catalase, superoxide dismutase, and glutathione peroxidase, and reducing glutathione content. These results indicated that FC alleviated glyco‐oxidation and oxidative damage in diabetes, consistent with its reported anti‐inflammatory properties [45].

**TABLE 2:** Other reported biological activities of fangchinoline.

Activity	Study type	Experimental model	Main findings	Ref
Antiglaucoma	In vivo	Swiss albino mice; unilateral laser‐induced ocular hypertension FC: 10/30 mg/kg i.p./1 week	FC ¯ high IOP improved glaucoma‐related markers FC restored aqueous humor SOD/MDA and improved vitreous glutamate FC ¯ retinal IL‐1β/IL‐6/TNF‐α, ‐RGC count, and ¯ microglia count FC ¯ retinal NF‐κB and TNF‐α mRNA	[44]

Antidiabetic	In vivo	Male albino rats; streptozotocin‐induced diabetes FC (100 or 200 mg/kg/oral/45 days)	FC attenuated diabetes‐linked oxidative injury FC reduced lipid peroxidation and ROS levels in the liver, kidney, and heart tissues FC improved antioxidant markers (SOD, catalase, Gpx, and GSH) FC ¯ raised glycoprotein (total hexoses, hexosamines, sialic acid, and fucose) FC improved liver and pancreatic histopathology in diabetic rats	[45]

Anti‐Sjögren’s syndrome	In vivo and in vitro	NOD/ShiLtj mice (SS model) CD^4+^ T cells and Jurkat T cells	FC alleviated SS‐like dry mouth and reduced salivary gland lymphocyte infiltration/foci, with fewer CD^4+^ T cells in infiltrating foci in vivo FC (1‐2 μM) inhibited the proliferation of activated CD4+ T cells in vitro	[46]
	In vivo and in vitro	Primary mouse CD4^+^ T cells; NOD/ShiLtJ mice	Inhibited CD4^+^ T‐cell proliferation; alleviated dry mouth and salivary‐gland lymphocytic infiltration and reduced CD4^+^ T cells in infiltrating foci	[47]
	In vivo	Nod/LtJ mice; FC 2 mg/kg, i.p., every other day for 4 weeks	Improved salivary flow; reduced submandibular‐gland lymphocytic infiltration and peripheral blood anti‐Ro/SSA levels FC decreased splenic Th1, Th2, Th17, and Tfh cells and increased Treg cells	[48]

Antiosteoarthritis	In vitro and in vivo	Primary mouse chondrocytes (IL‐1β stimulation); DMM‐induced OA in C57BL/6 mice; human OA cartilage culture	FC alleviated IL‐1β–induced inflammatory/catabolic responses in chondrocytes FC ¯ iNOS and MMP3 and modulated aggrecan to support ECM synthesis FC inhibited NF‐κB signaling by suppressing IKKα/β activity and reducing p65 nuclear localization FC ameliorated cartilage degeneration in DMM‐induced OA mice	[49]

Anti‐intracellular bacterial	In vitro and in vivo	*Salmonella*‐infected macrophages and mice	FC restricted intracellular *Salmonella* through NOS2‐dependent NO signaling and enhanced bacterial clearance in combination with a suboptimal dose of ciprofloxacin	[39]

### 3.7. Anti‐Sjögren’s Syndrome (anti‐SS)

SS is a systemic autoimmune disease that primarily affects the exocrine (salivary and lacrimal) glands, where lymphocytic infiltration and glandular dysfunction contributed to dry mouth and dry eyes [46]. The effect of FC was associated with modulation of both B‐ and T‐lymphocyte responses. FC inhibited proliferation, induced cell‐cycle arrest, and promoted apoptosis in Raji and Daudi B‐lymphoid cells through the modulation of Akt/mTOR signaling. FC inhibited proliferation, induced cell‐cycle arrest, and promoted apoptosis in Raji and Daudi B‐lymphoid cells through the modulation of Akt/mTOR signaling [46]. Subsequently, Shao et al. investigated the effects of FC on CD4^+^ T cells. In NOD/ShiLtJ mice, FC alleviated dry mouth and lymphocytic infiltration in the salivary glands and reduced CD4^+^ T cells within the infiltrating foci. FC (1‐2 μM) inhibited the proliferation of activated primary mouse CD4^+^ T cells. FC upregulated miR‐506‐3p and suppressed NFATc1 expression [47]. Recently, Niu et al. have examined the effects of FC on T‐cell subsets in Nod/LtJ mice [48]. FC improved salivary flow, reduced lymphocytic infiltration of the submandibular glands, and decreased circulating anti‐Ro/SSA antibody levels. It also reduced splenic Th1, Th2, Th17, and Tfh cells while increasing Treg cells. Target fishing coupled with mass spectrometry identified PKM2 as a potential molecular target, while molecular docking established FC–PKM2 interaction [48].

### 3.8. Anti‐OA Activity

He et al. investigated FC as a cartilage‐protective agent in OA. In vitro, FC alleviated IL‐1β–induced cartilage inflammation, lowered inducible nitric oxide synthase (iNOS) and matrix metalloproteinase‐3 (MMP3), and modulated aggrecan expression in a manner consistent with improved extracellular matrix homeostasis. FC ameliorated articular cartilage degeneration, restored aggrecan levels, and suppressed MMP3 and iNOS levels. Thus, FC induced its effect by modulating NF‐κB signaling, reducing IL‐1β‐induced phosphorylation of p65 and IKKα/β while increasing IκBα and limiting p65 nuclear localization [49].

### 3.9. Protective Effects Against Mitochondrial Oxidative Injury

Chen et al. further reported the protective effects of FC against mitochondrial oxidative stress using tBHP‐treated H9c2 cardiomyocytes [50]. FC (conc. 5 μM) preserved cell viability, reduced both cellular and mitochondrial ROS levels, improved ATP levels, and counteracted the tBHP‐induced loss of mitochondrial membrane potential. It also reduced DRP1 expression, suggesting its effect on the mitochondrial fission response associated with oxidative injury. Interestingly, tetrandrine showed closely similar protective potential, contributing their activity to the bisbenzylisoquinoline scaffold [50].

## 4. PKs, Bioavailability, and Safety of FC

FC has been assessed in several PK studies; however, the available evidence is mainly preclinical. Many studies focused on developing quantification and analytical methods for detecting FC following the administration of plant extracts or traditional formulations containing FC among their constituents (Table 3) [53, 58–60]. FC exhibited C_max_, T_max_, and t_1/2_ values that lined up with a relatively late absorption and elimination profile under the specified conditions [51, 53, 59].

**TABLE 3:** Reported pharmacokinetic, bioavailability, distribution, and safety data for FC.

FC/preparation/model	Concentration/route	PK/bioavailability findings	Distribution/safety findings	Related alkaloid comparison	Key limitation	Ref.
*Stephaniae Tetrandrae* Radix extract; rats	Extract 0.82 g/kg, oral	FC: C_max_ 84.56 ± 3.28 ng/mL; T_max_ 10.17 ± 3.04 h; t_1/2_ 24.29 ± 6.89 h. AUC_0–t_ 1551.05 ± 156.83; AUC_0–∞_ 1712.53 ± 159.44	—	Tetrandrine showed higher C_max_ and AUC than FC, while both tetrandrine and FC maintained higher concentrations for longer than cyclanoline	FC was administered as a constituent of a plant extract, not as isolated FC	[51]

70% aqueous‐ethanol extract of *Cyclea peltata*; Wistar rats	CP 50, 500, or 1000 mg/kg, oral; 28 days	—	FC and its decomposed mass were detected in liver and kidney, but not in the brain	Tetrandrine and its decomposed mass were detected in brain, liver, and kidney	Toxicity and distribution results relate to the whole *C. peltata* extract, not to isolated FC	[52]

Fangji Huangqi decoction (FHD); rats	FHD, oral	FC: T_max_ 9.67 ± 0.82 h; C_max_ 17.84 ± 2.61 ng/mL; AUC_0–t_ 635.4 ± 133.7 ng·h/mL; AUC_0–∞_ 864.4 ± 206.7 ng·h/mL; t_1/2_ 33.13 ± 10.68 h	—	Tetrandrine: T_max_ 8.00 ± 2.19 h; C_max_ 55.63 ± 8.32 ng/mL; AUC_0–t_ 1740 ± 498.8 ng·h/mL; AUC_0–∞_ 1999 ± 541.3 ng·h/mL; t_1/2_ 37.40 ± 5.62 h	Multicomponent traditional formula did not establish PK of isolated FC	[53]

*S. tetrandra* aqueous extract and isolated FC; rats plus in vitro binding/trapping assays	Oral and i.v. comparative administration	Whole‐blood exposure to FC was higher than plasma exposure. Oral exposure after the aqueous extract was higher than after isolated FC High plasma‐protein and erythrocyte binding and lysosomal trapping were demonstrated	—	Tetrandrine showed the same major qualitative features: greater whole‐blood than plasma exposure, matrix‐dependent exposure, blood‐component binding, and lysosomal trapping	The mechanism underlying the pharmacokinetic matrix effect was not established	[54]

Isolated FC, tetrandrine, and cepharanthine; PAMPA + cell‐based assays	In vitro	FC showed substantially lower passive membrane permeability than tetrandrine and cepharanthine. Permeability differed among isolated‐alkaloid, alkaloid mixture, and *S. tetrandra*‐extract conditions	Phospholipidosis occurred at higher alkaloid concentrations in cell assays	Tetrandrine and cepharanthine were significantly more permeable than FC	PAMPA estimated passive diffusion only and did not establish in vivo oral bioavailability, metabolism, or active transport	[28]

FC assessed computationally with other alkaloids	*In silico*	Predicted unbound fraction for FC was 0.323 Predicted total clearance was 0.70 log mL/min/kg FC was predicted not to inhibit the assessed CYP450 enzymes	FC was predicted as non‐hepatotoxic but caused AMES toxicity The selected alkaloids, including FC, were predicted not to inhibit hERG I but to inhibit hERG II	Computational comparison with several alkaloids; results varied among compounds across ADMET results	All ADMET findings were computational predictions and required experimental validation	[55]

Feiyanning formula (FYN); normal and LPS‐induced ALI BALB/c mice	FYN 0.715 g/kg, oral	ALI vs normal: FC plasma AUC_0–t_ 315.47 ± 22.81 ⟶ 573.99 ± 46.61 ng·h/mL; C_max_ 20.78 ± 5.19 ⟶ 35.42 ± 4.91 ng/mL; T_max_ 4 ⟶ 16 h; CLz/F 5.64 ⟶ 0.78 L/h/kg	In lung tissue, FC AUC_0–t_ and C_max_ were significantly lower in ALI mice despite the higher plasma exposure.	Tetrandrine showed a similar disease‐associated increase in plasma exposure T_max_ for both tetrandrine and FC was 16 h in ALI mice	FC was administered within a multicomponent formula	[56]

Isolated FC; rat liver microsomes (RLMs), human liver microsomes (HLMs), and male SD rats receiving abemaciclib	FC 50 mg/kg, orally before abemaciclib 30 mg/kg in rats	FC inhibited abemaciclib metabolism: IC_50_ 2.02 μM in RLM and 12.24 μM in HLM; mixed inhibition in RLM (Ki 1.84 μM) and competitive inhibition in HLM (Ki 6.52 μM) In rats, FC ↑ abemaciclib AUC_0–∞_ 1.54‐fold and ↓ CLz/F to 37.63%	FC significantly affected the metabolism of abemaciclib	—	Inhibition type differed between RLM and HLM	[57]

Isolated FC; male Sprague–Dawley rats (PK) and Kunming mice (acute toxicity)	PK: FC 10 mg/kg, i.p.; acute toxicity: single oral gavage	t_1/2_ 6.68 ± 1.91 h; T_max_ 1.75 ± 0.27 h; C_max_ 218.10 ± 37.12 ng/mL; AUC_0–t_ 1164.23 ± 165.68 h·ng/mL; AUC_0–∞_ 10,021.59 ± 3797.88 h·ng/mL; MRT 8.46 ± 2.14 h	Oral LD_50_: 2060.23 mg/kg in males and 1481.93 mg/kg in females No mutagenic activity was observed in the Ames test	—	PK testing used i.p. administration and measured plasma only; intestinal FC exposure was not determined	[25]

Isolated FC; C57BL/6 male mice	15 mg/kg, i.p., for 5 days	—	No significant changes in ALT, AST, urea, or creatinine compared to vehicle controls. Histopathological analysis of liver, kidney, and spleen revealed no detectable toxicity at the tested dose	—	Small safety cohort (*n* = 3/group), short exposure period, males only	[39]

*Note:* FC, fangchinoline; FYN, Feiyanning formula; AUC, area under the concentration–time curve; C_max_, maximum concentration; T_max_, time to Cmax; t_1/2_, elimination half‐life; CLz/F, apparent clearance.

Abbreviations: ALI, acute lung injury; HLM, human liver microsomes; MRT, mean residence time; PAMPA, parallel artificial membrane permeability assay; RLM, rat liver microsome.

PK studies of FC have largely involved plant extracts or traditional multicomponent formulations rather than isolated FC. Following oral administration of Fangji Huangqi decoction (FHD), FC showed slow absorption and elimination under the tested conditions [53]. Other studies demonstrated that the PK behavior of FC differed according to the administered preparation, including *S. tetrandra* extracts and Fangji Huangqi Tang [51, 59]. Lyu et al. investigated the PKs of the major components of FHD following oral administration in rats. FC was found to enter the bloodstream and show slow absorption and elimination, with a long residence time, while it did not show dose‐dependent absorption [42]. However, these findings should be interpreted within the context of multicomponent preparations rather than isolated FC.

Physicochemical and in silico analyses by Shah et al. provided predicted physicochemical, drug‐likeness, and ADMET characteristics of FC; however, these computational findings require experimental validation [51, 55, 59]. Recently, Hu et al. have evaluated FC PKs following a single intraperitoneal administration of 10 mg/kg in male Sprague‐Dawley rats [25]. The analysis was limited to plasma concentrations and therefore may not reflect FC exposure in intestinal tissue, where TGEV replication occurs. Acute oral toxicity testing in Kunming mice yielded LD_50_ values of 2060.23 mg/kg in males and 1481.93 mg/kg in females, while FC showed no mutagenic activity under the experimental conditions of the Ames test [25]. Sadhu et al. further reported that FC administered intraperitoneally at 15 mg/kg for 5 days did not significantly alter ALT, AST, urea, or creatinine, with no detectable toxicity in liver, kidney, or spleen histopathology under the tested conditions, suggesting that FC is well tolerated in vivo at this dose [39].

Anuja et al. assessed the tissue distribution of FC following repeated oral administration of a 70% aqueous‐ethanol extract of *Cyclea peltata* in Wistar rats [52]. LC–Q–TOF–MS analysis detected FC and its decomposed mass in the liver and kidney, but not in the brain, whereas tetrandrine and its decomposed mass were detected in the brain, liver, and kidney. Since the plant extract was administered, these findings did not establish the tissue distribution of isolated FC [52].

Liao et al. showed that FC exposure in whole blood was higher than that measured in plasma [54]. Also, the systemic exposure after oral administration of a *S. tetrandra* aqueous extract was higher than that observed after oral administration of isolated FC, indicating a PK matrix effect. In vitro experiments further showed high plasma protein binding, and extensive erythrocyte binding of FC. FC was also subject to lysosomal trapping, which was reduced by the lysosomal inhibitor [54]. These findings indicated that plasma protein binding, erythrocyte binding, and lysosomal trapping can markedly reduce the free systemic exposure of FC [54]. Comparative PK studies with the related BBAs tetrandrine showed broadly similar slow absorption and elimination profiles, although tetrandrine exhibited higher systemic exposure than FC under the same formulation conditions [51, 53].

The plasma exposure to FC was found to be higher in LPS‐induced ALI mice than in normal mice following the oral administration of the Feiyanning formula [56], while FC exposure in lung tissue was significantly lower in ALI mice. These findings demonstrated differences in the PK behavior of FC between normal and ALI mice; however, FC was administered as a constituent of the multicomponent Feiyanning formula, not as an isolated compound [56]. Chen et al. reported that FC inhibited abemaciclib metabolism in both rat and human liver microsomes, showing mixed competitive/noncompetitive inhibition in rat liver microsomes and competitive inhibition in human liver microsomes. In rats, FC pretreatment increased the AUC_0_–∞ of abemaciclib to 1.54‐fold of the control and decreased CLz/F by 37.63%, whereas Tmax, Cmax, and t_1_/_2_ were not significantly changed. These findings suggest a potential PK interaction between FC and abemaciclib [57]. The different inhibition patterns observed in rat and human liver microsomes should be considered when interpreting the translational relevance of these findings. Overall, the available findings provided preliminary information on the PKs, tissue distribution, safety, and potential drug interactions of FC, but remain limited to preclinical and in silico studies. Interpretation is further complicated by the frequent assessment of multicomponent plant extracts and traditional formulations rather than isolated FC. Human PKs, absolute oral bioavailability, detailed metabolism, tissue distribution after the administration of isolated FC, and clinically relevant drug–drug interactions remain insufficiently characterized. Further studies using isolated FC, standardized PK designs, comprehensive ADMET evaluation, and ultimately clinical investigation are required to define its translational potential.

## 5. Structure–Activity Relationship

Available data regarding the structure–activity relationship for FC are limited, and a definitive antiviral SAR has not yet been established. Khadilkar et al. reported that the lower passive membrane permeability of FC relative to the structurally related BBAs tetrandrine and cepharanthine may be attributed to its additional free hydroxyl group [28]. A study by Sadhu et al. revealed that C7 hydroxyl group modification may affect antiviral activity. It was found that the 7‐O‐methanesulfonate derivative MK‐04‐003 had improved antiviral activity compared with FC (Sadhu et al. 2023). This highlighted the C7 hydroxyl group as a relevant position for further investigation of the physicochemical and antiviral properties of FC.

## 6. Challenges in Clinical Development and Translation

The translation of FC from preclinical research to clinical investigation continues to be challenging, as the current evidence has not sufficiently determined the relationship between the obtained results from experimental studies and clinically adequate compound concentration in humans. Although recent studies provided additional PK, safety, and mechanistic information regarding FC, these data are derived from various experimental conditions and are still not sufficient to determine safe and effective compound levels in humans. Moreover, evidence obtained with herbal formulations containing FC cannot be directly applied to the isolated compound. Therefore, before assessing the therapeutic potential of FC in humans, it is critically important to establish clinically relevant compound levels, confirm the consistency of the reported findings, and establish an accurately characterized safety profile.

## 7. Conclusion

FC was reported to display diverse bioactivities across different disease models, particularly in infectious diseases, based on studies published between 2021 and August 2026. The reported findings suggested that FC can modulate various signaling pathways, including oxidative stress and immune responses, highlighting its multitarget potential.

Additionally, it has been reported to exhibit antiviral activity, particularly against coronaviruses (e.g., SARS‐CoV‐2) and flaviviruses, including ZIKV, through mechanisms involving the inhibition of viral entry and internalization, modulation of endolysosomal trafficking and autophagy, and modulation of innate immune responses. Antimicrobial activity was reported against *Candida* species and intracellular bacterial pathogens, including *Salmonella*. It also demonstrated protective effects against metabolic diseases, such as diabetes, through antioxidant and anti‐inflammatory mechanisms. The hepatoprotective, antifibrotic, anti‐OA, anti‐SS, and neuroprotective properties of FC further support its diverse biological profile. However, despite these promising activities, most findings are based on preclinical investigations, predominantly in vitro, with relatively few in vivo studies. In addition, variations in concentrations, experimental models, and study designs restricted direct comparisons. Significant gaps remain, particularly in PK characterization, bioavailability, toxicological evaluation, drug–drug interactions, and structure–activity relationship studies. Although recent investigations provided additional data on systemic exposure, tissue distribution, safety, and potential PK interactions, the available evidence remains preclinical. Human PKs, absolute oral bioavailability, detailed metabolism, and tissue distribution following the administration of isolated FC remain insufficiently characterized.

Furthermore, no clinical investigations have been reported yet, and the relationship between in vitro activity and in vivo efficacy remains to be clearly established. Available antiviral SAR studies also remain limited, although the modification of the C7 hydroxyl group provided preliminary information for further structural optimization. Future research should focus on well‐designed in vivo studies with standardized methodologies, comprehensive PK and safety evaluations, and further elucidation and experimental validation of the molecular mechanisms. In addition, the optimization of FC derivatives, exploration of rational combination strategies, and suitable formulation strategies should be further investigated to determine their effects on the physicochemical and PK properties of FC and its derivatives. Overall, FC represents a potential natural product scaffold; however, further studies are required to determine whether its reported biological activities can be translated into clinically meaningful applications, particularly in infectious diseases.

**Nomenclature**
AD: Alzheimer’s disease
ADAM17: A disintegrin and metalloproteinase 17
ADMET: Absorption, distribution, metabolism, excretion, and toxicity
Akt: Protein kinase B
ALI: Acute lung injury
ALT: Alanine aminotransferase
AMPK: AMP‐activated protein kinase
ASFV: African swine fever virus
AST: Aspartate aminotransferase
ATP: Adenosine triphosphate
AUC: Area under the concentration–time curve
BBAs: Bisbenzylisoquinoline alkaloids
BEAS‐2B: Human bronchial epithelial cell line
CAT: Catalase
CC_50_
: 50% cytotoxic concentration
CD4^+^
: Cluster of differentiation 4–positive T cells
CETSA: Cellular thermal shift assay
CLz/F: Apparent clearance
Cmax: Maximum plasma concentration
COX‐2: Cyclooxygenase‐2
CPE: Cytopathic effect
DARTS: Drug affinity responsive target stability
DENV: Dengue virus
DRP1: Dynamin‐related protein 1
EC_50_
: 50% effective concentration
ECM: Extracellular matrix
EMCV: Encephalomyocarditis virus
EMT: Epithelial–mesenchymal transition
ERBB2: Erb‐B2 receptor tyrosine kinase 2
FAK: Focal adhesion kinase
FC: Fangchinoline
GSH: Reduced glutathione
GSH‐Px: Glutathione peroxidase
H1N1: Influenza A virus subtype H1N1
H3N2: Influenza A virus subtype H3N2
hACE2: Human angiotensin‐converting enzyme 2
HFMD: Hand, foot, and mouth disease
HLM: Human liver microsomes
HSV‐1: Herpes simplex virus type 1
IC_50_
: 50% inhibitory concentration
IFN: Interferon
IKK: IκB kinase
IL: Interleukin
iNOS: Inducible nitric oxide synthase
i.p.: Intraperitoneal
ISGs: Interferon‐stimulated genes
JEV: Japanese encephalitis virus
JNK: c‐Jun N‐terminal kinase
LDH: Lactate dehydrogenase
MDA: Malondialdehyde
MDCK: Madin–Darby canine kidney
MERS‐CoV: Middle East respiratory syndrome coronavirus
MFC: Minimum fungicidal concentration
MIC: Minimum inhibitory concentration
MMP: Matrix metalloproteinase
MRT: Mean residence time
mTOR: Mechanistic target of rapamycin
NAC: N‐acetyl‐L‐cysteine
NFATc1: Nuclear factor of activated T cells 1
NF‐κB: Nuclear factor kappa B
NO: Nitric oxide
NOS2: Nitric oxide synthase 2
Nrf2: Nuclear factor erythroid 2–related factor 2
OA: Osteoarthritis
PAMPA: Parallel artificial membrane permeability assay
PAMs: Porcine alveolar macrophages
pAPN: Porcine aminopeptidase N
PEDV: Porcine epidemic diarrhea virus
PI3K: Phosphoinositide 3‐kinase
PKM2: Pyruvate kinase M2
PRRSV: Porcine reproductive and respiratory syndrome virus
RLM: Rat liver microsomes
ROS: Reactive oxygen species
SAR: Structure–activity relationship
SARS‐CoV: Severe acute respiratory syndrome coronavirus
SARS‐CoV‐2: Severe acute respiratory syndrome coronavirus 2
SI: Selectivity index
SOD: Superoxide dismutase
SRC: SRC proto‐oncogene, nonreceptor tyrosine kinase
SS: Sjögren’s syndrome
STING: Stimulator of interferon genes
t_1_/_2_
: Elimination half‐life
Tfh: T follicular helper cells
TFEB: Transcription factor EB
TGEV: Transmissible gastroenteritis virus
TGF‐β: Transforming growth factor beta
Th1: T helper 1 cells
Th2: T helper 2 cells
Th17: T helper 17 cells
Tmax: Time to maximum concentration
TNF‐α: Tumor necrosis factor alpha
Treg: Regulatory T cells
vRNPs: Viral ribonucleoproteins
VSV: Vesicular stomatitis virus
ZEB1: Zinc finger E‐box‐binding homeobox 1
ZIKV: Zika virus

## Author Contributions

Alaa Sirwi analyzed the data, prepared figures and/or tables, and authored or reviewed drafts of the paper. Hagar M. Mohamed analyzed the data, prepared figures and/or tables, and authored or reviewed drafts of the paper. Gamal A. Mohamed conceived and designed the idea, analyzed the data, prepared figures and/or tables, and wrote the draft of the paper. Basma G. Eid analyzed the data, prepared figures, funding acquisition, project administration, and authored or reviewed drafts of the paper. Hossam M. Abdallah analyzed the data and authored or reviewed drafts of the paper. Sabrin R. M. Ibrahim conceived and designed the idea, analyzed the data, prepared figures and/or tables, and wrote the draft of the paper.

## Funding

The authors gratefully acknowledge the Deanship of Scientific Research (DSR) at King Abdulaziz University, Jeddah, Saudi Arabia, for funding this project under Grant No. IPP: 1248‐249‐2026 and for providing technical support.

## Disclosure

All authors approved the final draft.

## Conflicts of Interest

The authors declare no conflicts of interest.

## Data Availability

Data sharing is not applicable to this article as no datasets were generated or analyzed during the current study.
